# Point-of-care ultrasound evaluation and puncture simulation of the internal jugular vein by medical students

**DOI:** 10.1186/s13089-018-0115-2

**Published:** 2018-12-19

**Authors:** José Muniz Pazeli, Ana Luisa Silveira Vieira, Rosimary Souza Vicentino, Luisa Jabour Pazeli, Bernardo Costa Lemos, Marinna Marques Rodrigues Saliba, Pedro Andrade Mello, Maurício Dutra Costa

**Affiliations:** 1 FAME - Barbacena’s School of Medicina, Barbacena, Brazil; 2 Federal University of Juiz de Fora, Juiz de Fora, Brazil; 3 SUPREMA - School of Medical Sciences and Health of Juiz de Fora, Juiz de Fora, Brazil

**Keywords:** Central venous catheterization, Medical education, Point-of-care ultrasound

## Abstract

**Objectives:**

To show that medical students can evaluate the internal jugular vein (IJV) and its anatomical variations after rapid and focused training. We also aimed to evaluate the success rate of IJV puncture in simulation following traditional techniques (TTs) and monitored via ultrasound (US).

**Materials and methods:**

Six medical students without experience with US were given 4 h of theoretical–practical training in US, and then evaluated the IJV and common carotid artery (CCA) of 105 patients. They also simulated a puncture of the IJV at a demarcated point, where a TT was theoretically performed.

**Results:**

Adequate images were obtained from 95% of the patients; the IJV, on the right side, was more commonly found in the anterolateral position in relation to the CCA (38%). On the left side, the most commonly position observed was the anterior (36%). The caliber of the IJV relative to the CCA greatly varied. The success rate in the IJV puncture simulation, observed with US, by the TTs was 55%.

**Conclusion:**

The training of medical students to recognize large neck vessels is a simple, quick, and feasible task and that can be integrated into the undergraduate medical curriculum.

## Introduction

Central venous access is the cannulation of a central vein via percutaneous puncture [1]. It is indicated in certain medical situations such as the hemodynamic monitoring of critical ill patients and the infusion of medications, parenteral nutrition, or hemodialysis [2].

Central puncture is typically performed at the jugular, subclavian, or femoral vein [3]. In practice, the internal jugular vein (IJV) is a common site, but recent data show that subclavian puncture is associated with lower rates of infection and thrombosis [4].

Traditionally, puncture of a central vein is performed as the “blind” insertion of a needle following anatomical landmarks [5]. Puncture of the IJV for central venous access was first described in 1969 [6]. Since then, various other approaches have been proposed: anterior, central, and posterior approaches [7, 8].

While these traditional techniques (TTs) based on anatomical markers have been validated, they are associated with failure rates ranging from 5 to 40% [3] and complications such as pneumothorax, hematoma, hemothorax, lesions of nerves, and others. These occur at rates between 5 and 19% [9].

For these reasons, ultrasound (US) has been recommended since the 1980s to guide the procedure, optimizing successful cannulation and minimizing complications [10].

Over the last two decades, US equipment has become progressively more compact and accessible, facilitating its use at the point of care. Point-of-care ultrasound (POCUS) is performed and interpreted by the physician in charge wherever the patient is located [11]. Therefore, US is actually an extension of the physical examination, offering the physician a valuable complement to the patient’s propedeutic and monitoring in addition to guiding procedures, thus increasing their safety and efficacy [12, 13].

The use of US for central venous access can considerably reduce its risks and complications [5, 14].

Ultrasound-guided techniques (USGTs) can be quickly assimilated by professionals who have no previous experience. Moureau et al. suggested that supervised training ideally spans 16 to 18 h, although the technique has been mastered in less time [15, 16].

In this study, we aimed to show that medical students can evaluate the IJV and its anatomical variations after rapid and focused training. We also aimed to evaluate the success rate of IJV puncture in simulation following TTs and monitored via US.

## Methods

### Study design

This cross-sectional, observational study used humans and was approved by the Research Ethics Committee from Fundação Hospitalar do Estado de Minas Gerais (FHEMIG) under Protocol Number 59842616.0.0000.5119.

### Study settings and population

From November 2016 to April 2017, we enrolled 105 patients hospitalized in the Clinic, Gynecology, and Obstetrics wards at Santa Casa de Misericórdia de Barbacena—Minas Gerais Brazil.

Patients aged at least 18 years of either sex were included. Each indicated agreement to participate in the study by providing their signature or their guardian’s signature on the Informed Consent Form.

Excluded were patients with prior central venous access and those who did not agree to participate. Each patient was stable, without critical disease, and denied a prior central venous access.

### Study protocol

Six 4th-year medical students without experience with US were selected from the Barbacena’s School of Medicine and SUPREMA-Faculty of Medical Sciences and Health of Juiz de Fora. They were given 4 h of theoretical–practical training in US coordinated by an intensivist and nephrologist with extensive experience in TTs and USGTs.

The training included four 60-min stages: (a) a theoretical exposition including TTs, the physical principles of US, and USGTs; (b) a practical demonstration of anatomical markers and positioning the transducer; (c) practical training in ultrasonographic visualization of vessels and simulation of puncture via TTs, using each other as models; and (d) four supervised examinations per student and completion of the report.

Ultrasonographic evaluations were performed using a Sonosite Fujifilm MTurbo portable US (Bothell, WA, USA) equipped with a linear transducer with a frequency between 6 and 13 MHz.

**Fig. 1:**
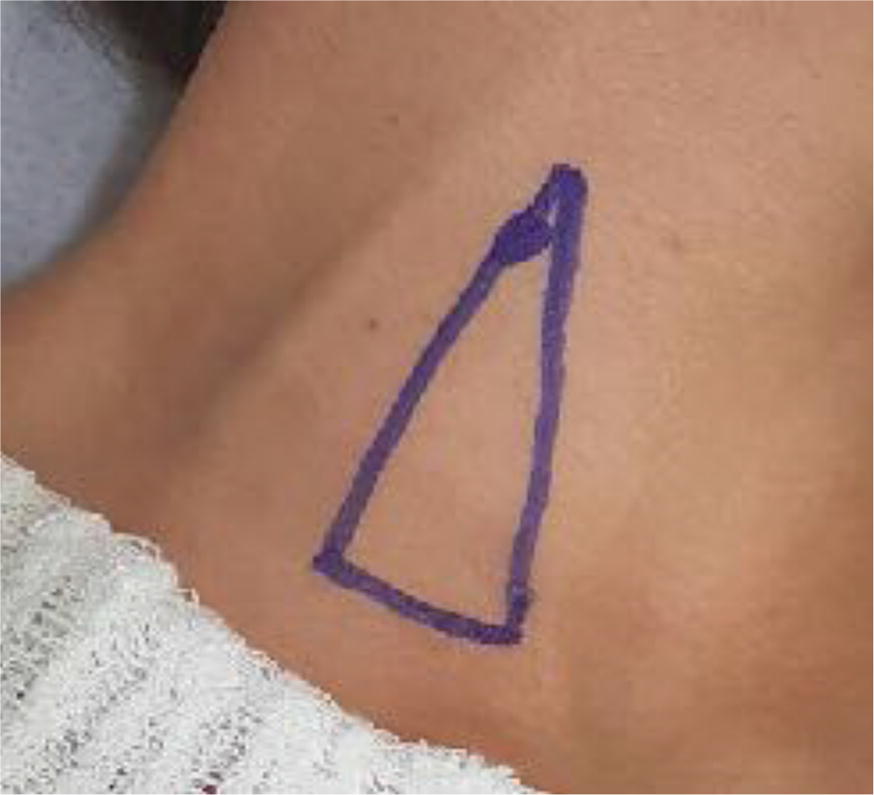
Identification of the TT puncture point

Patients were placed in dorsal decubitus at 0º, and the cervical region was exposed via contralateral rotation of the neck. The apex of the triangle, composed of the clavicle and the two borders of the sternocleidomastoid muscle, was determined. The pulse at the common carotid artery (CCA) was verified, and immediately lateral to that point, the site chosen for the TT was marked with a pen (Fig. 1).

**Fig. 2:**
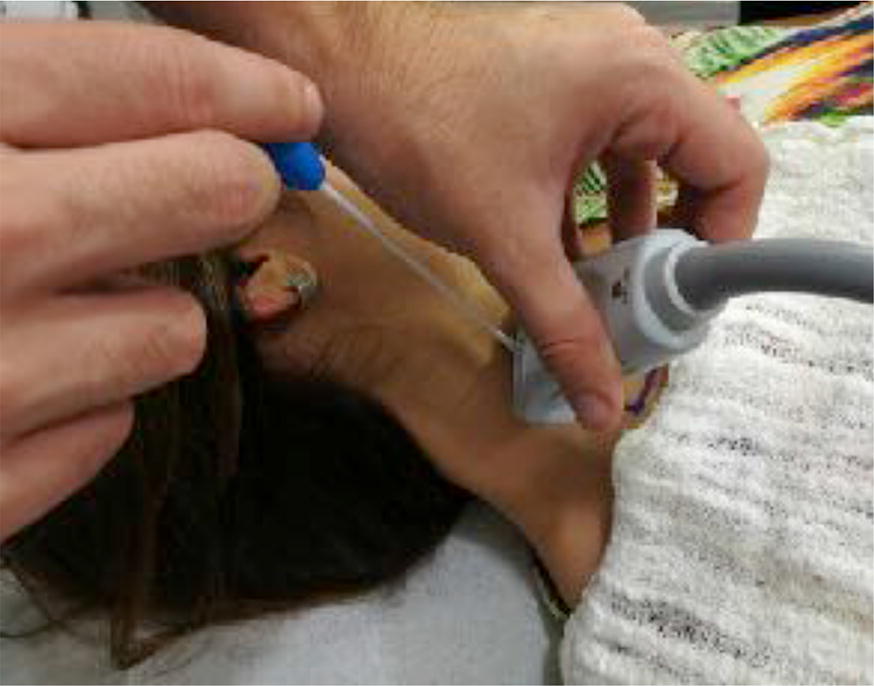
Simulation of puncture

**Fig. 3:**
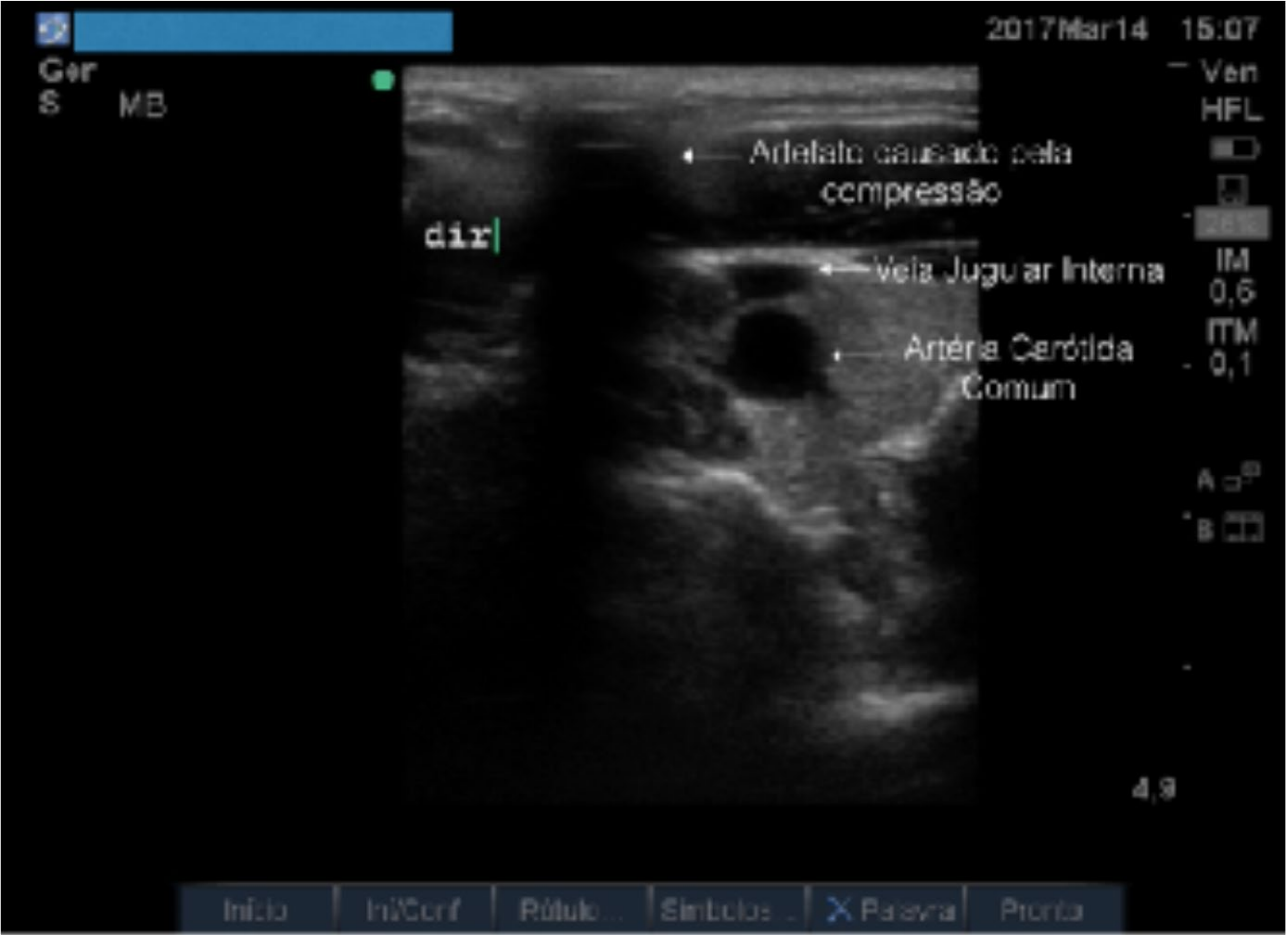
Observation of the CCA, IJV and the artifact caused by the compression of the point where the puncture would be performed by the TT. In this case, the puncture would not reach the IJV

The US evaluation began by positioning the transducer at the marked point. Then, puncture via a TT was simulated by positioning a flexible cotton-coated rod at the marked point (Fig. 2). The artifact caused by the slight compression of this puncture point was visualized on the US screen and an imaginary line between that point and the position of the patient’s ipsilateral nipple was drawn. This line corresponds to the path the needle would make in an actual puncture guided by anatomical landmarks. If this line passed through the IJV, it was understood that puncture via a TT would have been effective. If the traced line did not cross the vein path, the TT was deemed a failed attempt (Fig. 3). All exams and simulations were filmed and, shortly thereafter, they were analyzed by two physicians with extensive experience in both puncture techniques.

The evaluation was fully qualitative, and the following variables were obtained: IJV position relative to the CCA (medial, anteromedial, lateral, anterolateral, posterior, or anterior); visual estimation of the IJV caliber relative to the CCA (less than 50%, between 50 and 100%, 101 and 150%, 150 and 200%, or greater than 200%); probability of reaching the IJV via the TT relative to the relative size and position of the IJV (success or failure).

### Data analysis

Sample size was calculated based on an assumption that more than 12% of the Western population presents significant anatomical variations of the great vessels of the neck, as reported in the literature [17]. Thus, for a power of 80%, with a confidence index of 95%, the minimum sample was 63 individuals [18].

Relationships between studied variables were determined using the Fisher exact test and the Chi square test. Differences were considered significant when *p *< 0.05. Accuracies and their confidence intervals were calculated. Statistical analysis was performed using Stata software, version 9.2 (StataCorp, College Station, TX, USA).

## Results

Of 105 patients ranging in age from 18 to 89 years, 82 were women.

Five were excluded because of poor image quality, leaving 100 patients included in the analysis.

On the right side, the IJV was most commonly found in the anterolateral position relative to the carotid artery (38% of the time) and was also found anterior (31%), anteromedial (21%), or lateral (10%) to that artery. In no case was it found in the medial position (Table 1).

**Table 1:** Location of IJV in relation to CCA

	Right side of neck			Left side of neck		
	*N*	%	CI	*N*	%	CI
Anterior	31	31	(21.9–40.0)	36	36	(26.5–45.4)
Anterolateral	38	38	(28.4–47.5)	31	31	(21.9–40.0)
Lateral	10	10	(13.0–28.9)	1	1	(0.90–2.90)
Anteromedial	21	21	(4.1–15.80)	31	31	(21.9–40.0)
Medial	0			1	1	(0.90–2.90)
Posterior	0			0		

*IJV* internal jugular vein, *CCA* common carotid artery, *CI* confidence interval

On the left side, it was most commonly found in the anterior position (36%) and was also found in the anterolateral (31%), anteromedial (31%), lateral (1%), and medial (1%) positions (Table 1). On both sides, the posterior position and its variables were not found.

The caliber of the IJV relative to the CCA greatly varied, as shown in Table 2.

**Table 2:** Size of IJV in relation to CCA

	Right side *n* = 100			Left side *n* = 100			Total *n* = 200		
	*n*	%	CI	*n*	%	CI	*n*	%	CI
Collapsed	20	20	(12.1–27.8)	18	18	(10.4–25.5)	38	19	(31.2–44.7)
< 50%	7	7	(1.9–12.0)	10	10	(4.1–15.8)	17	8.5	(11.7–22.2)
50–100%	28	28	(19.1–36.8)	27	27	(18.2–35.7)	55	27.5	(48.1–61.8)
101–150%	11	11	(4.8–17.1)	5	5	(0.7–9.2)	16	8	(10.9–21.0)
151–200%	12	12	(5.6–18.3)	10	10	(4.1–15.8)	22	11	(16.2–27.7)
> 200%	22	22	(13.8–30.1)	30	30	(21.0–38.9)	52	26	(45.0–58.9)
Total	100	100		100	100		200	100	

*IJV* internal jugular vein, *CCA* common carotid artery, *CI* confidence interval

The probability of reaching the IJV for puncture was related to its size and anatomical position relative to the CCA (Tables 3 and 4).

**Table 3:** Probability of reaching IJV in relation to the relative size of IJV to CCA

	Yes		No	
	*n*	%	*n*	%
Collapsed	15	12	23	30.67
< 50%	10	8	7	7
50–100%	29	23.2	26	26
101–150%	13	10.4	3	4
151–200%	16	12.8	6	6
> 200%	42	33.6	10	10
Total	125	100	75	100

*IJV* internal jugular vein, *CCA* common carotid artery

Chi square = 21.723, *p* = 0.001

**Table 4:** Probability of reaching IJV in relation to its location in relation to CCA

	Yes		No	
	*n*	%	*n*	%
Anterior	42	62.69	25	37.31
Anterolateral	43	62.32	26	37.68
Anteromedial	35	67.31	17	32.69
Lateral	5	45.55	6	54.45
Medial	0	0	6	6
Total	125	100	75	100

*IJV* internal jugular vein, *CCA* common carotid artery

Fischer test *p* = 0.478

Several results were noted when the right and left sides of a given patient were compared. One was the number of times the IJV was in a position opposite to the anatomical (medial or anteromedial) position on one side yet in the anatomical (lateral or anterolateral) position on the opposing side. Of the 21 patients in which the vein was anteromedial to the artery on the right side, 19% of the time, it was in the anatomical position on the left side. Of the 32 patients in which the vein was anteromedial or medial on the left side, 28% of the time, it was in an anatomical position on the right side (Table 5).

**Table 5:** Relationship between anatomical position of IJV and CCA in the right side

Right/left	Anterior		Anterolateral		Anteromedial		Lateral		Medial		Total	
	*n*	%	*n*	%	*n*	%	*n*	%	*n*	%	*n*	%
Anterior	11	35.5	8	25.8	12	38.7	0	0	0	0	31	100
Anterolateral	16	42.1	15	39.5	6	15.8	1	2.6	0	0	38	100
Anteromedial	6	28.6	4	19.0	11	52.4	0	0	0	0	21	100
Lateral	3	30.0	4	40.0	2	20.0	0	0	1	10	10	100
Medial	0	0	0	0	0	0	0	0	0	0	0	0
Total	36		31		31		1		1		100	100

*IJV* internal jugular vein, *CCA* common carotid artery

The size of the vein relative to the artery in the same patient was examined. Of 20 patients in which the IJV was collapsed on the right side, it was not collapsed on the left side 45% of the time. Similarly, of 18 patients in which it was collapsed on the left side, it was not collapsed on the right side 43% of the time (Table 6).

**Table 6:** Relationship between size of the IJV in relation to CCA in the right (row) and in the left side (column) of the neck

Right/left	Collapsed		< 50%		50–100%		101–150%		151–200%		> 200%		Total	
	*N*	%	*N*	%	*N*	%	*N*	%	*N*	%	*N*	%	*N*	%
Collapsed	11	55	3	15.0	6	30.0	0	0.0	0	0.0	0	0.0	20	100
< 50%	3	42.8	3	42.8	1	14.3	0	0.0	0	0.0	0	0.0	7	100
50–100%	4	14.3	3	10.7	9	32.1	3	10.7	2	7.1	7	25.0	28	100
101–150%	0	0.0	0	0.0	3	27.7	2	18.9	3	27.3	3	27.3	11	100
151–200%	0	0.0	0	0.0	5	41.6	0	0.0	3	25.0	4	33.3	12	100
> 200%	0	0.0	0	0.0	3	13.6	0	0.0	2	9.1	16	72.7	22	100
Total	18		10		27		5		10		30		100	

*IJV* internal jugular vein, *CCA* common carotid artery

## Discussion

These data show that medical students can be trained to recognize the anatomy of large vessels in the neck using US after rapid and focused training. Adequate images were obtained from 95% of the patients chosen for evaluation, reinforcing the simplicity and feasibility of the method.

Puncture of the IJV is a procedure often performed in hospitals, emergency departments, and during pre-hospital care for a wide range of indications. Although several studies have shown that USGTs are cost-effective, not only for reducing mechanical complications and infections, but primarily for increasing the success rate and reducing procedure time, it is not used routinely, especially in developing countries [14, 19], given the lack of equipment availability, lack of trained professionals, and other reasons [16].

**Fig. 4:**
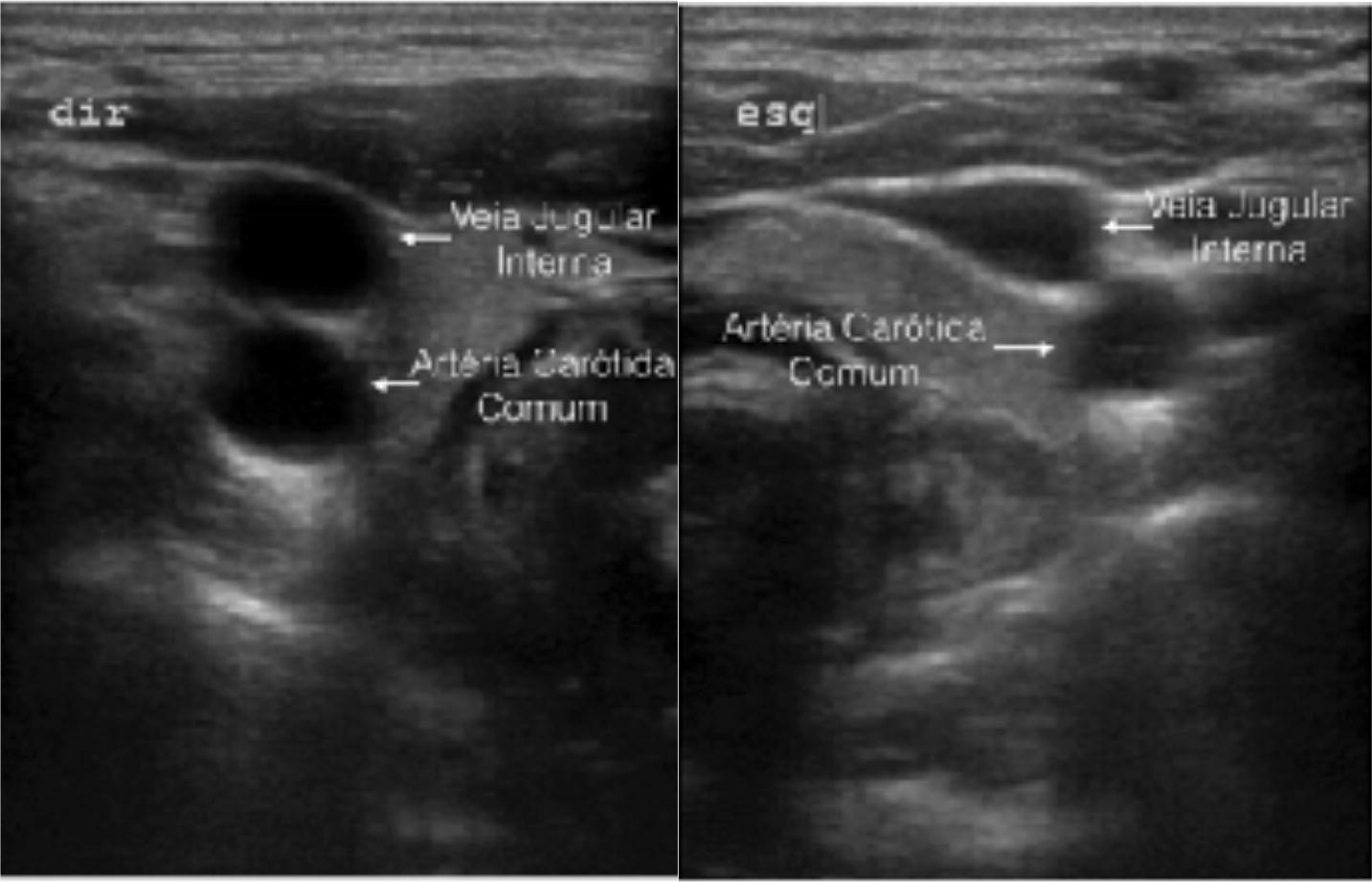
Right and left IJV of a same patient, anterior and medial to the ACC, respectively

As found by several others [20–23], great anatomical variability is found between the IJV and the CCA. Variability is also seen between the left and right sides of the same individual (Fig. 4). The IJV is in an anatomical position 48% of the time on the right side and 32% of the time on the left side. Use of TTs assumes that the IJV is in an anatomical position on both sides, but this was observed in fewer than 50% of our patients, combining anterolateral and lateral positions.

**Fig. 5:**
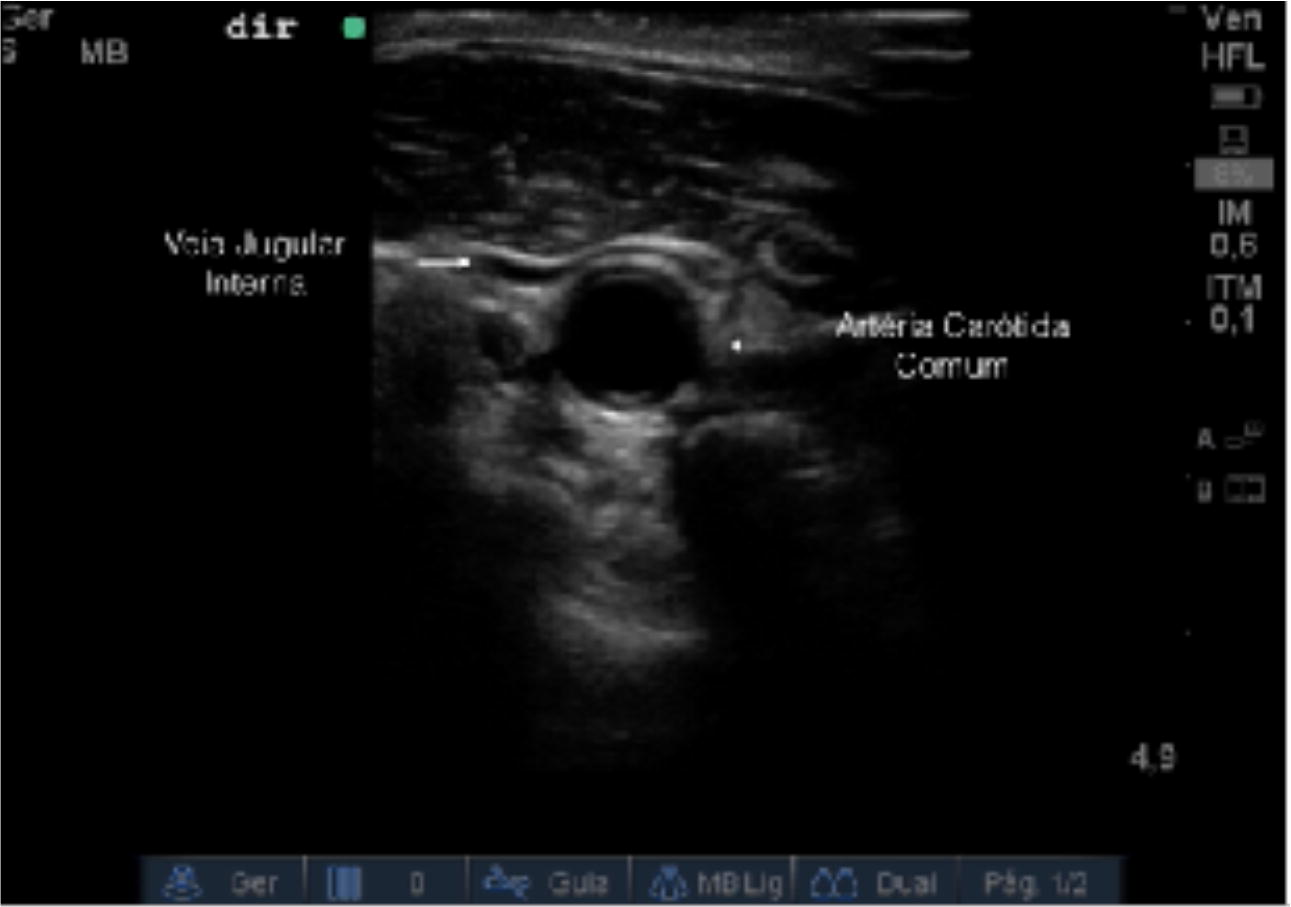
Right IJV completely collapsed, anterolateral to CCA

Additionally, great variability was found in the caliber of the IJV in our sample, and this is also supported by the literature [17, 21]. In our sample, the IJV was completely collapsed on the right and left sides 20% and 18% of the time, respectively (Fig. 5). Puncturing a collapsed vein is nearly impossible, even if the needle is directed correctly. Another complicating factor is that, in our sample, the IJV was anterior to the CCA 68% of the time. Attempting puncture in this condition can have disastrous consequences because of the risk of reaching the CCA, given that vein collapse amplifies this risk.

We emphasize that in our patients, examination was conducted in a supine position with 0° slope despite the recommendation to position the patient in a Trendelenburg, a suggestion that is not always feasible for a variety of reasons. In this study, contralateral rotation of the neck was used because this position is most frequently recommended in IJV puncture by TTs. However, it has been shown that neck rotation does not interfere with the rate of success and/or reduction of complications when using USGTs [24].

In this study, puncture of the IJV was simulated at the demarcated point, where a TT was theoretically performed (Fig. 1). The success rate using US was determined as well (Fig. 3). Our data show that puncture could reach the IJV 62.5% of the time. Therefore, in 37.5% of the patients (75 of 200 simulations), more than one puncture attempt would be performed, increasing the possibility of complications [25]. The IJV was collapsed in 15 of 125 simulations in which the needle was supposed to reach that vein. Because it is unlikely that puncture is successful with a collapsed vein, we can conclude that the success rate would be 55% for the first attempt, confirming results from studies comparing TTs to USGTs [14, 19].

As suggested by Dinh et al. [26], incorporating training in USGTs in the curriculum for undergraduate medical students is highly desirable and feasible, and this is confirmed by our data. Implementing this and other applications of POCUS in medical schools would ensure that new generations of physicians are naturally empowered, increasing the resoluteness and safety of patient care.

This study is limited by the simulated nature of our procedures; however, actual puncture is not possible for ethical reasons. Also, students performed these procedures after training and without direct supervision, but our objective was to precisely verify the feasibility and simplicity of ultrasonographic visualization of the vessels of the neck after a short period of training. To avoid misinterpretation of the images, all were recorded and reviewed by physicians experienced in POCUS.

## Conclusions

These data suggest that training medical students to recognize large neck vessels is a simple, quick, and feasible task and that can be integrated into the undergraduate medical curriculum.

## Abbreviations

CCA: common carotid artery
IJV: internal jugular vein
POCUS: point-of-care ultrasound
TTs: traditional techniques
US: ultrasound
USGTs: ultrasound-guided techniques
